# Is MELD‐Na Truly Predictive of Diuretic Response in Heart Failure? Methodological and Physiological Considerations

**DOI:** 10.1002/clc.70300

**Published:** 2026-04-17

**Authors:** Karan Chaman Lal, Minal Fatima, Sana Soomro, Aiza Siddiqui

**Affiliations:** ^1^ Department of Medicine Liaquat University of Medical & Health Sciences Jamshoro Pakistan; ^2^ Department of Medicine Allama Iqbal Medical College Lahore Pakistan; ^3^ Department of Medicine Jinnah Sindh Medical University Karachi Pakistan; ^4^ Department of Medicine Islamic International Medical College Rawalpindi Pakistan

**Keywords:** acute heart failure, cardiorenal syndrome, diuretic response, MELD‐Na score, natriuresis

Dear Editor,

We read with keen interest the retrospective observational study by Kozluca et al., which evaluated the role of MELD score in predicting the diuretic response in patients with heart failure [1]. The study provides valuable insights into the cardiorenal interaction by measuring the urinary sodium to assess the link between MELD score and early diuretic response in this population. However, there are a few methodological limitations that need to be addressed to improve the reliability and generalizability of the results.

A key limitation in this study is that urine sodium is the primary factor affecting the diuretic response, whereas MELD‐Na includes serum sodium and INR, which reflect sodium handling and neurohormonal activation. Because both the predictor (MELD‐Na) and the outcome (diuretic response) are influenced by sodium‐related factors, the apparent correlation is circular reasoning rather than an independent predictive relationship. Testani et al. defined the diuretic response as measured solely by the total sodium excretion in urine. Salt retention is the primary mechanism that drives extracellular volume expansion [2]. They present a physiology‐based strategy for assessing natriuretic response to loop diuretics and emphasize urine salt as a clinical sign of diuretic efficacy.

Furthermore, this study does not fully adjust for well‐known factors that influence diuretic response in patients with acute decompensated heart failure. Although the authors describe an independent link between the MELD‐Na score and poor diuretic response, several key confounders were not adequately considered. These include the starting dose of loop diuretics, underlying kidney function, the intensity of neurohormonal therapy, and the timing of intravenous diuretic administration. All of these factors are known to directly affect natriuresis and urine sodium‐based measures of diuretic response [3, 4, 5]. Diuretic response in heart failure is inherently multifactorial, reflecting the combined effects of renal function, neurohormonal activation, haemodynamics, and pharmacologic exposure. Failure to adjust for these variables limits causal interpretation and introduces significant residual confounding. This concern is particularly relevant given that patients with poorer diuretic response received higher doses of loop diuretics, which themselves are associated with impaired natriuresis and worse outcomes, independent of congestion severity. Prior studies have shown that dose‐adjusted or dose‐normalized natriuresis is essential to distinguish true diuretic resistance from treatment intensity [3, 4]. The absence of such modeling substantially undermines the internal validity of the reported findings [5].

Another critical limitation in this study is that the MELD‐Na score was measured solely at the time of admission. However, diuretic response in heart failure is an evolving process that changes continually. Renal adaptation and decongestive therapy can change the cardiorenal and hepatorenal congestion. Therefore, measuring it at only one point in time limits its reliability and validity. Another retrospective observational study solved this problem by measuring the MELD‐Na scores at the time of admission, along with at days 2–3 of hospital stay, showing that a serial assessment of this score provides better prognostic information as compared to baseline value only [6]. Therefore, measuring the MELD‐Na scores periodically would improve the validity of the results and could enhance their applicability to a larger population.

In conclusion, although this study adds to the literature by addressing the relationship between MELD‐Na and diuretic response, these methodological limitations should be noted. Using a physiologically based strategy for analyzing diuretic response can significantly improve the reliability of the findings. Additionally, there should be an appropriate adjustment of confounding variables and serial assessment of MELD‐Na. Only through the application of all these methodological strategies can the association between MELD‐Na scores and diuretic response be established and used in clinical practice.

## Author Contributions

Conceptualization: Karan Chaman Lal. Initial Draft: Karan Chaman Lal. Literature Review: Karan Chaman Lal, Minal Fatima, Sana Soomro, Aiza Siddiqui. Writing, Reviewing, and Editing: Karan Chaman Lal, Minal Fatima, Sana Soomro, Aiza Siddiqui. Final Approval: Karan Chaman Lal, Minal Fatima, Sana Soomro, Aiza Siddiqui.

## Funding

The authors have nothing to report.

## Conflicts of Interest

The authors declare no conflicts of interest.

## Data Availability

The authors have nothing to report.
